# Network pharmacology-based investigation and experimental validation of the mechanism of scutellarin in the treatment of acute myeloid leukemia

**DOI:** 10.3389/fphar.2022.952677

**Published:** 2022-09-07

**Authors:** Zhe Huang, Yan Yang, Xianming Fan, Wenzhe Ma

**Affiliations:** ^1^ State Key Laboratory of Quality Research in Chinese Medicine, Macau University of Science and Technology, Macau, China; ^2^ Department of Pediatrics, The Affiliated Hospital of Southwest Medical University, Sichuan Clinical Research Center for Birth Defects, Luzhou, China; ^3^ Department of Respiratory and Critical Care Medicine, Affiliated Hospital of Southwest Medical University, Luzhou, China

**Keywords:** Scutellarin, AML, network pharmacology, JNK/Caspase-3 signal pathway, apoptosis

## Abstract

**Background:** It has been demonstrated that scutellarin, a natural flavone compound from *Scutellaria lateriflora* and *Scutellaria barbata*, exerts selective cytotoxicity against a range of cancer cells. However, the underlining mechanism of scutellarin on acute myeloid leukemia (AML) remains elusive.

**Methods:** In this study, the combination of network pharmacology and experimental verification was performed to identify the pharmacological mechanisms of scutellarin for AML therapy. The public databases, such as PharmMapper, UniProt, OMIM, GeneCards, DrugBank and PharmGkb database, were used to sceen the potential targets of scutellarin and AML. The protein-protein interaction (PPI), gene ontology (GO) and Kyoto Encyclopedia of Genes and Genomes (KEGG) pathway enrichment analysis were conducted to uncover the mechanism of scutellarin in the treatment of AML. Finally, the network pharmacological results were further confirmed by *in vitro* and *in vivo* experiments.

**Results:** First and foremost, we totally obtained 289 target genes for scutellarin and 10998 disease targets for AML. 253 overlapping genes were preliminarily considered the potential targets of scutellarin for AML treatment. The results of PPI network analysis, GO analysis and KEGG pathway enrichment demonstrated that the anti-AML effect of scutellarin may focused on MAPK signaling pathway. Furthermore, the cytologic tests suggested that scutellarin can inhibit AML cells proliferation through the mediation of JNK/Caspase-3 pathway. Meanwhile, pretreatment with the JNK inhibitor SP600125 rescued scutellarin-induced apoptosis. Similarly, scutellarin obviously suppressed subcutaneous xenograft growth in nude mice *via* regulating the JNK/Caspase-3 signaling pathway.

**Conclusion:** In this study, we integrated network pharmacology-based prediction and experimental validation and revealed the importance of the JNK pathway in scutellarin-mediated AML treatment.

## Introduction

Acute myeloid leukemia (AML), a typical blood cancer, is featured by changes in hematopoietic cells, such as blocked differentiation, increased proliferation, or accumulation of blasts (Dohner et al., 2017). As the most common acute leukemia in adults, AML is diagnosed with a median age of 68 years and increasing with age (Reedijk et al., 2019; Ari Pelcovits 2020). Because the incidence trend is not optimistic, AML has high morbidity and mortality, which has become the focus of current research.

The ″3 + 7″ regimen, 3 days of daunorubicin followed by 7 days of cytarabine, is the standard treatment for AML. The overall survival rate in patients with AML under this standard regimen is only 40%, and the 5-years survival rate (5y-SR) of patients (≥60 years) is even lower, less than 10–15% (M.Heuser, 2020). In the past 30 years, with the rapid development of genomics, treatment methods for AML have gradually diversified. Hematopoietic stem cell transplantation, molecular targeted therapy and, more recently, CAR-T cell immunotherapy have been used in the clinic (Sallman et al., 2018; Takami, 2018; Daver et al., 2019; Yilmaz & Daver, 2019; Becker & Fathi, 2020; Roberts, 2020). Despite significant improvement in the treatment of AML, the 5y-SR has not improved significantly (Dohner et al., 2015; Thomas & Majeti, 2017). Therefore, safer and more effective treatments are urgently needed.

Traditional Chinese Medicine (TCM) has been widely used in clinical for thousands of years and is one of the most abundant resources for the discovery of anticancer drugs. As is well known, TCM treated diseases in a holistic concept and possessed the characteristics of multi-target and multi-approach, which coincides with the prevention and treatment strategy of cancer (Xiang et al., 2019). For a long time, TCM has played a unique advantage in tumor treatment. A number of clinical trials have confirmed that the combination of active ingredients of TCM and chemotherapies can prolong the survival period, improve the survival status, and can also enhance the detoxification effect (Huang X. M et al., 2020). Many Western countries also use TCM as complementary and alternative therapy (Luo et al., 2014). Scutellarin is a natural flavone compound from *Scutellaria barbata* and *Scutellaria lateriflora,* herbs that have a long history in TCM (Wang & Ma, 2018). Research on scutellarin over the past 3 decades has accumulated a wealth of evidence confirming its effectiveness in the treatment of cerebrovascular and cardiovascular diseases, especially coronary heart disease and ischemic stroke (Chen et al., 2020; Xu et al., 2021). In the past 10 years, scutellarin has been reported to have selective cytotoxicity against cancer cells from different tissues, such as glioblastoma, gastric, colorectal, and lung cancer (Fu Li and niu, 2021; Liu et al., 2019; Zeng et al., 2021; Zhang et al., 2021). Various mechanisms of action have been reported for the anticancer activities of scutellarin. Scutellarin can downregulate the hedgehog signaling pathway and thus achieve the inhibition of the proliferation and migration in human colorectal cancer cells (Tan et al., 2020). It has been reported that scutellarin could induce apoptosis through the regulatuion of TGF-β1/smad2/ROS/Caspase-3 pathway in A549 cells (Zhang et al., 2021), and via STAT3 pathway in HepG2 cells (Xu & Zhang, 2013). Scutellarin can also suppress metastasis and chemoresistance in glioma cells (Tang et al., 2019). However, the anticancer effect of scutellarin on AML has not been reported so far.

Accompanying with the quick development of system biology and bioinformatics, network pharmacology provides a new method for revealing the complex mechanism of TCM and the interrelationships among drugs, targets and diseases (Huang et al., 2020). In line with TCM theory which emphasizes the synergistic effect of ingredients, network pharmacology closely observes the effects of drugs on disease.

**FIGURE 1:**
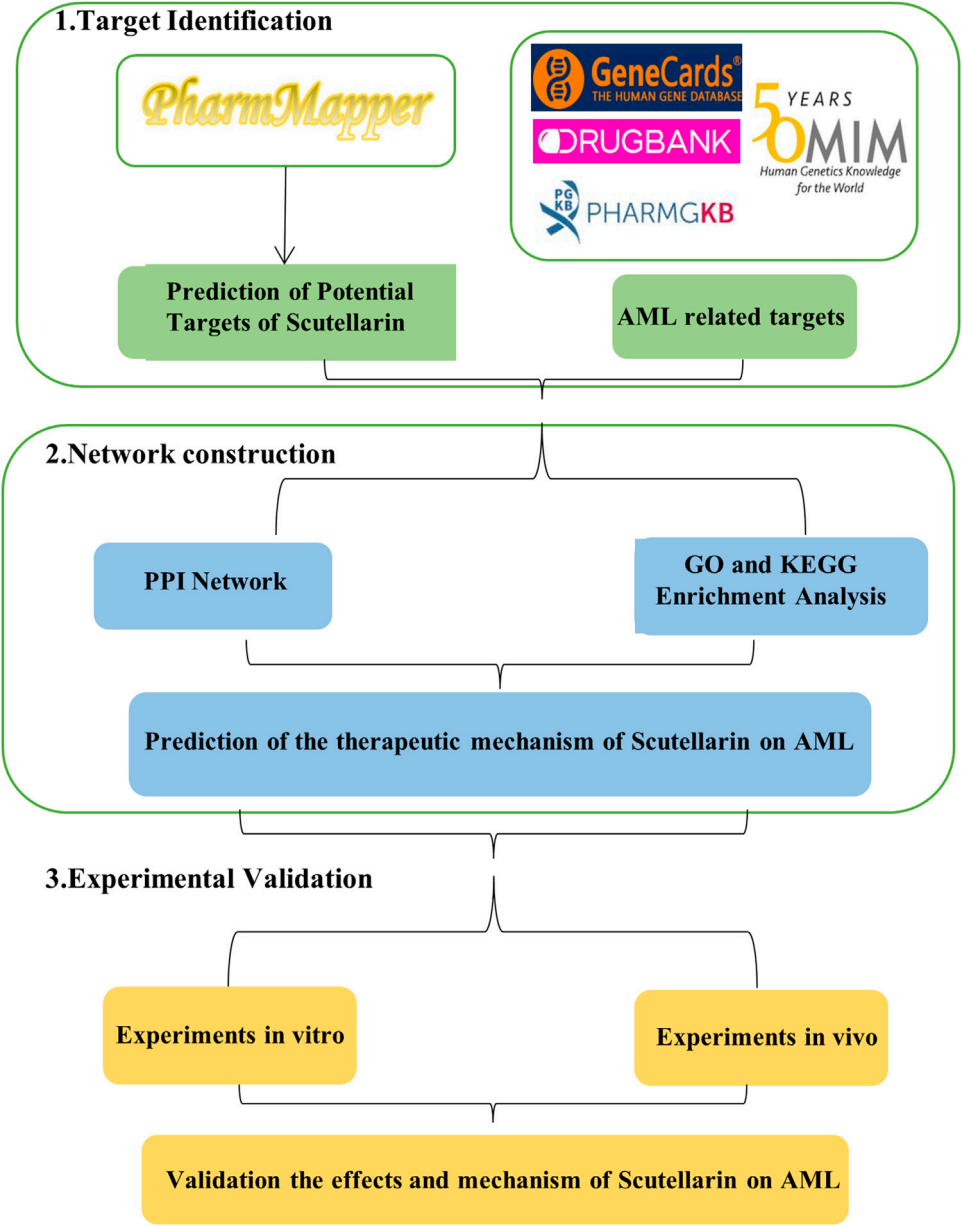
The workflow of action mechanism of scutellarin on treating AML in this study.

In our study, network pharmacology was employed to elucidate the molecular mechanisms of scutellarin for AML therapy, which was confirmed by *in vitro* and *in vivo* experimental validation. The workflow is shown in Figure 1.

## Materials and methods

### Cell culture

Human AML cell lines (HL-60, KG-1) were obtained from the US Model Culture Collection (ATCC, US). THP-1 human AML cells were purchased from the Chinese Academy of Sciences Cell Bank. THP-1, HL-60, KG-1 cells were cultured in RPMI-1640 medium (Gibco) supplemented with 10% fetal bovine serum (FBS, Gibco), 1% penicillin (pen) and streptomycin (strep).

### Chemicals

Scutellarin (CAS#27740-01-8) was provided by Weiqi Biological Technology Co. Ltd. Breviscapine was provided by Yunnan Plant Pharmaceutical Co. Ltd. (National drug approval Z53020121). Z-VAD (OMe)-FMK (Z-VAD, HY-16658, MCE), SP600125 (S1876, Beyotime), CCK-8 kit (Japan Tongren Chemical Research Institute), Annexin V-FITC Apoptosis Detection Kit I (556547, BD Bioscience).

### Antibodies

Western blot immunoassay was performed with antibodies against p-JNK(Cat.#9251S), JNK (Cat.#9252S), cleaved caspase-3(Cat.#9664), cleaved PARP (Cat.#5625), anti-rabbit secondary antibody (Cat.#7074) and anti-mouse secondary antibody (Cat.#7076). The above antibodies were all provided by Cell Signaling Technology. Αnti-β-actin (Cat.#A5441) was provided by Sigma Aldrich.

### Proliferation assay *in vitro*

Cell proliferation assay was assessed with the CCK-8 kit (Japan Tongren Chemical Research Institute) on leukemia cell lines. HL-60 (2×10^4^ cells/well), THP-1 (1.5×10^4^ cells/well) and KG-1 (1.5×10^4^ cells/well) cells were inoculated in 96-well plates at a volume of 90 μL/well. Then 10 μl medium containing various doses of scutellarin was added and incubated for 72 h at 37°C. Next, 5 μl/well CCK-8 solution was added and incubated for 4 h at 37°C. The SpectraMax 190 microplate reader (Molecular Devices) was used to measure the absorbency at 450 nm. The relative cell growth rate was calculated with the following equation: Relative Growth (%) = [OD (treated) -OD (blank)]/[OD (control)-OD (blank)] ×100%. The IC_50_ of scutellarin was counted by Graph Pad Prism 8.0 software.

### The targets of scutellarin in AML

The PubChem database (https://pubchem.ncbi.nlm.nih.gov/) was applied to obtained chemical structure of scutellarin. The PharmMapper database (http://www.lilab-ecust.cn/pharmmapper/) was utilized to generate targets with high scores. Then, the protein target names were converted to corresponding gene symbols in UniProt database (http://www.uniprot.org/). The disease targets of AML were retrieved from OMIM, GeneCards, DrugBank and PharmGkb databases. The targets associated with AMLwere obtained by merging the targets of the four databases. Finally, the predicted targets of scutellarin against AML were collected by intersection of the drug and disease targets.

### Construction of a “drug-disease-target” network

Based on the above analyses, the potential target genes of scutellarin were matched with the disease targets of AML to obtain the common targets. The network diagram between the scutellarin and AML-related targets was established from Cytoscape 3.8.0 (http://www.cytoscape.org/).

### PPI network

The PPI network was constructed by importing the targets of scutellarin against AML to the STRING database (https://string-db.org/). Then, the interaction information was further visually analysed by Cytoscape3.8.0. In the PPI network, the key genes based on degree scores were screened using the Cytohubba plug-in.

### GO and KEGG pathway analysis

GO and KEGG enrichment pathway analysis were used to search the key targets of scutellarin against AML by using the Bioconductor package in R (4.1.1) language. Statistical significance was set at q value ≤ 0.05.

### Apoptosis assay

HL-60 cells were seeded in 6-well plates at 5×10^5^ cells/well and treated with scutellarin of indicated concentrations. Then cells were centrifuged in the tube after 24 h, rinsed twice with pre-cold PBS buffer, then incubated with 5 µl PI and 5 µl FITC Annexin V in 100 µl 1×Binding Buffer at room temperature in the dark for 20 min. Next, the cells were resuspended in 400 μl 1×Binding Buffer. Cellular apoptosis was assessed with Annexin V-FITC Apoptosis Detection Kit I (BD Bioscience). The cells were analyzed by a flow cytometer (BD Bioscience) after passing through a filter. The data were analyzed by FlowJo 7.6 software.

### Western blotting

HL-60 cells were inoculated in 6-well plates and incubated in a medium containing scutellarin for indicated time point. Cell lysates were prepared with RIPA buffer supplemented with phosphatase inhibitor (Roche) and protease inhibitor cocktail (Roche). Protein concentrations were quantified by the BCA protein assay kit (P0012, Beyotime). Protein samples were heated at 95°C for 10 min following diluted in 5 x loading buffer. Then, 30 µg of each sample was fractionated on SDS-PAGE gels, transferred to PVDF membrane (Millipore) and incubated with different primary antibodies overnight at 4 C. Next, HRP-conjugated secondary antibodies (1:10000) were added at room temperature and the membranes were washed with 1×TBST. Proteins were visualized with SuperSignal West Pico Chemiluminescent Substrate or SuperSignal West Dura Extended Duration Substrate (Thermo Scientific).

### Xenograft assay

All mice used in the animal experiments were 4-6 weeks old female nude mice. 5×10^6^ HL-60 cells were inoculated subcutaneously into both hind limbs of nude mice. we randomly divided the mice into two groups: vehicle (normal saline, po), Breviscapine (60 mg/kg/d, po) when tumor volumes reached 100 mm^3^. The therapy lasted for 21 days and the tumor size and body weight were monitored every other day. The tumor volumes were calculated as 

Volume = (width^2^ × length)/2 (Huang et al., 2021).

All animal studies were approved by the Animal Ethical Committee of Affiliated Hospital of Southwest Medical University.

### Statistical analyses

All data were shown as mean ± standard deviation (SD). And statistical analyses were performed through Graph Pad Prism 8.0 software. The data were analyzed by one-way analysis of variance (ANOVA) if comparing between more than two groups. The student’s *t*-test was selected to compare two groups. * represents *p* < 0.05, ** represents *p* < 0.01, *** represents *p* < 0.001, **** represents *p* < 0.0001.

## Results

### The precidiction of anti-AML targets of scutellarin

**FIGURE 2:**
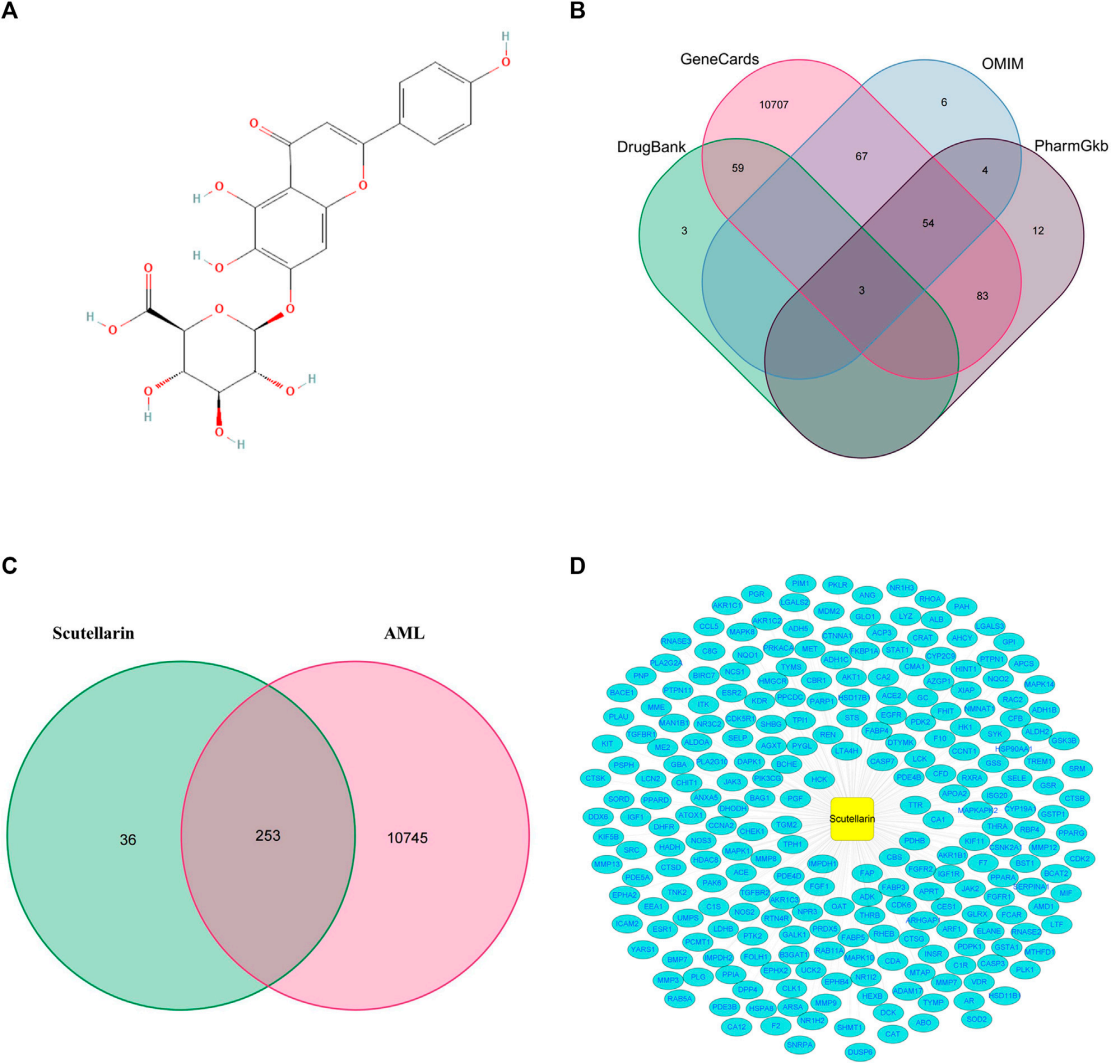
The precidiction of anti-AML targets of scutellarin. **(A)** Chemical structure of scutellarin. **(B)** The collection of AML-related genes obtained from four databases. **(C)** The intersection of AML-related genes and target genes of scutellarin. **(D)** The network map of “drug-disease-target”.

A total of 289 potential targets for scutellarin were retrieved from the PharmMapper database by querying the structure of scutellarin (Figure 2A; Supplementary Table S1). Similarly, 10998 targets related to AML were found in OMIM, GeneCards, DrugBank, and PharmGkb databases (Figure 2B; Supplementary Table S2). Subsequently, 253 genes were enriched after intersecting the targets of scutellarin with AML-related targets (Figure 2C; Supplementary Table S3). Finally, to elucidate the mechanism of scutellarin in AML treatment, a “drug-disease-target” network was constructed applying Cytoscape 3.8.0 software (Figure 2D).

### Analysis on AML-related PPI

**FIGURE 3:**
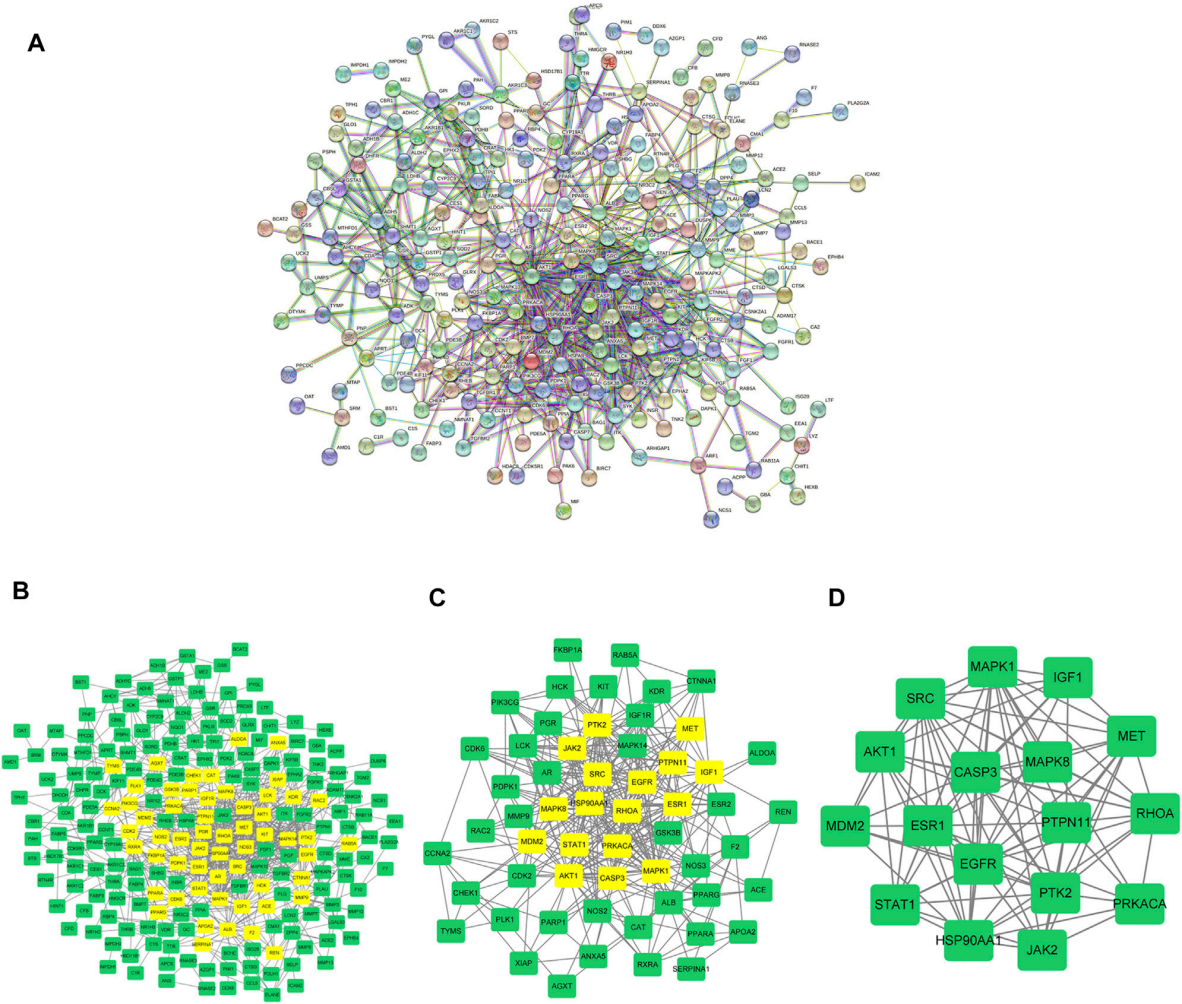
Analysis on AML-Related PPI. **(A)** The construction of protein interaction network of AML target genes induced by scutellarin. **(B)** The interactive PPI network of AML-related targets and scutellarin targets. **(C)** Further screening of AML target genes acted by scutellarin. **(D)** Seventeen core candidate target genes of scutellarin for AML treatment.

To analyze the protein-protein interaction network, we interrogated the 253 target genes, with high confidence set to 0.7, in the STRING database (Figure 3A; Supplementary Table S4). The core parts of the network were then analyzed by CytoNCA and finally three core topological networks were obtained, which contained 17 core genes, including MAPK8, CASP3, PRKACA, MAPK1, AKT1, HSP90AA1, MET, ESR1, SRC, EGFR, JAK2, IGF1, PTK2, STAT1, RHOA, MDM2, PTPN11 (Figures 3B–D; Supplementary Table S5).

### GO and KEGG pathway analysis

**FIGURE 4:**
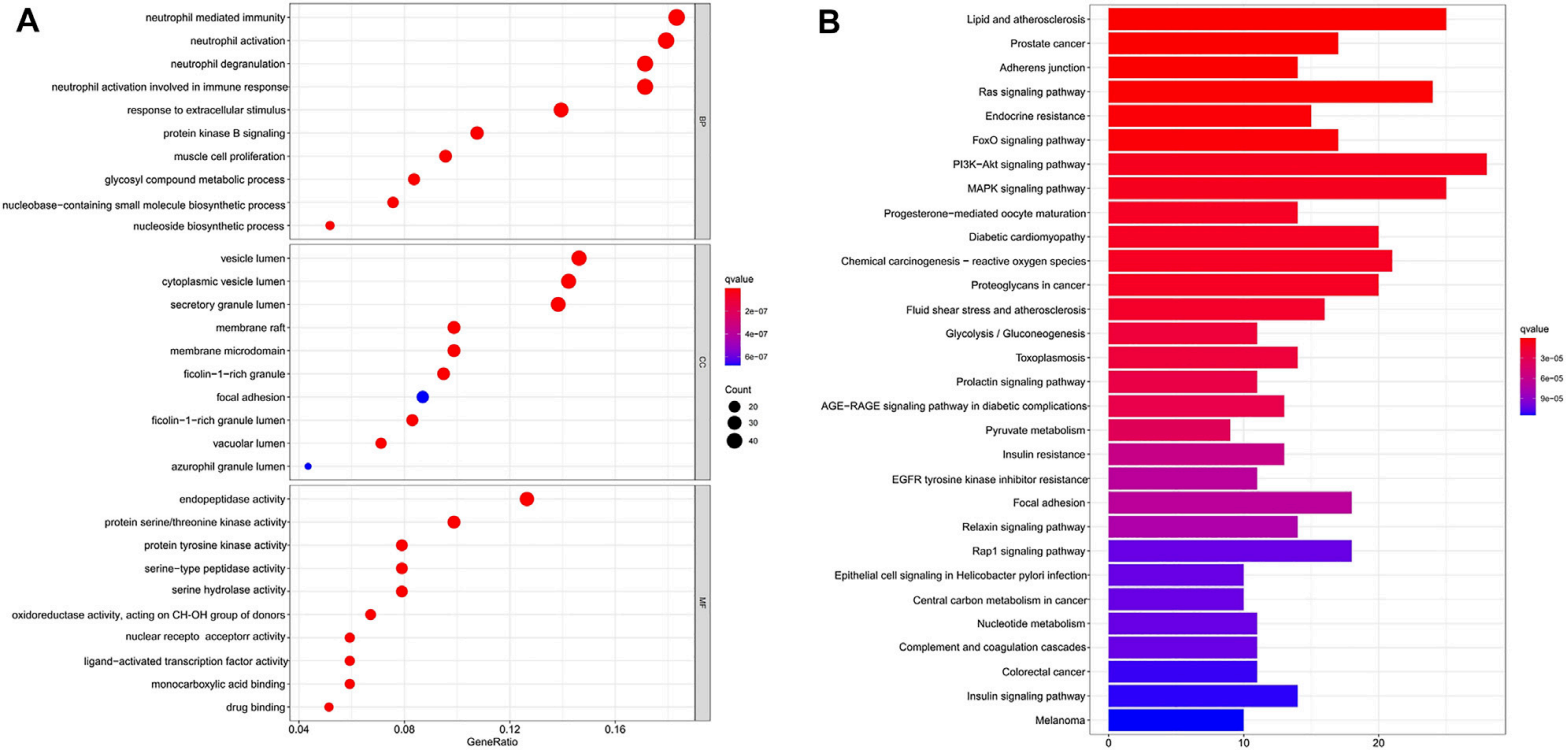
GO and KEGG pathway enrichment analysis. **(A)** GO analysis enrichment of candidate target genes of scutellarin for AML treatment. **(B)** KEGG pathways enrichment of candidate target genes of scutellarin against AML. (*p* < 0.05).

To gain more insights into the cellular function of scutellarin in AML cells, we performed a GO term enrichment analysis of the 253 targets. A total of 2201 GO terms were obtained. Among them, there are 1935 entries for biological process (BP), 52 entries for cellular composition (CC), and 214 entries for molecular function (MF). The top-10 significantly enriched terms in each category are shown in Figure 4A. Major terms in the BP category included neutrophil mediated immunity, response to extracellular stimulus, response to nutrient levels, cellular response to chemical stress, response to oxidative stress, and protein kinase B signaling. Major CC terms involved vesicle lumen, cytoplasmic vesicle lumen, secretory granule lumen, and membrane raft. Major MF terms covered protein serine/threonine kinase activity, protein tyrosine kinase activity, and serine-type peptidase activity. Regulation of the execution phase of apoptosis and cellular component disassembly in the execution phase of apoptosis were both enriched (Supplementary Table S6).

KEGG pathway enrichment analysis of the 253 targets enriched in 150 signaling pathways. It verified AML-related pathways involving the PI3K-Akt signaling pathway (hsa04151), MAPK signaling pathway (hsa04010), and Ras signaling pathway (hsa04014) (Supplementary Table S7). The top 30 KEGG pathways with high counts were shown in Figure 4B. The MAPK signaling pathway, which enriched a large count among AML-related pathways (Torres et al., 2010; Bomfim et al., 2019; Tian et al., 2019), plays a key role in the treatment of AML. Hence, a crucial branch of MAPK signaling, JNK pathway, was tested *in vitro* and *in vivo* to confirm whether it contributed to the anti-AML effects of scutellarin by inducing apoptosis.

### Scutellarin inhibits the growth of AML cells

**FIGURE 5:**
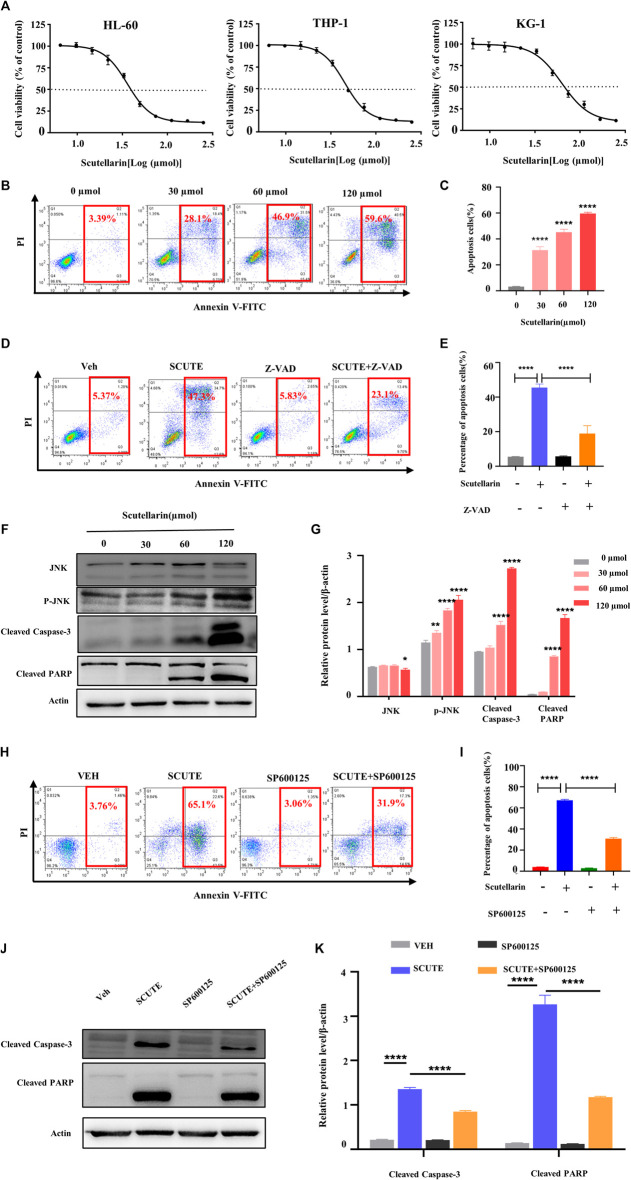
Scutellarin inhibits the growth of AML cells. **(A)** HL-60, THP-1 and KG-1 cells were treated with different concentrations of scutellarin for 72 h. Cell viability (% of control) was performed by CCK-8 assay. **(B)** Apoptosis analysis was measured by flow cytometry in HL-60 cells treated with different concentrations of scutellarin. **(C)** The statistical analysis result of **(B)**. **(D)** Apoptosis analysis was performed by flow cytometry in HL-60 cells incubated with vehicle or scutellarin (60 µM) for 24 h following pretreated with vehicle or Z-VAD-FKM (50 µM) for 2 h. **(E)** The statistical analysis result of **(D)**. **(F)** The levels of JNK, p-JNK, cleaved caspase-3 and cleaved PARP in HL-60 cells after scutellarin treatment by Western blot analysis. **(G)** The statistical analysis result of **(F)**. **(H)** Apoptosis analysis was performed by flow cytometry in HL-60 cells incubated with vehicle or scutellarin (60 µM) for 24 h following pretreated with vehicle or SP600125 (10 µM) for 2 h. **(I)** The statistical analysis result of **(H)**. **(J)** Western blot analysis of proteins in HL-60 cells incubated with vehicle or scutellarin (60 µM) for 24 h following pretreated with vehicle or SP600125 (10 µM) for 2 h. **(K)** The statistical analysis result of **(J)**. All data were analyzed as mean ± SD (*n* = 3, **p < 0.01, ***p < 0.001, ****p < 0.0001).

To investigate the cytotoxic effects of scutellarin, we treated AML cell lines for 72 h with various concentrations of scutellarin, and CCK-8 assay was employed to test the cell viability. As illutrated in Figure 5A, scutellarin dose-dependently reduced the viability of HL-60, THP-1, and KG-1 cells. The IC50 values of scutellarin on three AML cell lines were 35.99, 45.26, and 62.61 µM, respectively.

To confirm the molecular mechanism of scutellarin on AML cells, we focused on HL-60 cells, which was more sensitive to scutellarin relative to other cell lines. The pro-apoptotic effect of scutellarin on HL-60 cells was determined by flow cytometric analysis. After 24 h treatment with scutellarin at indicated concentrations, the apoptosis in HL-60 cells obviously increased in a dose-dependent manner (Figures 5B,C). Furthermore, pretreatment with Z-VAD-FMK (a pan-caspase inhibitor) can partially rescue apoptosis induced by scutellarin (Figures 5D,E), strongly indicating that scutellarin induced apoptosis in HL-60 cells.

**FIGURE 6:**
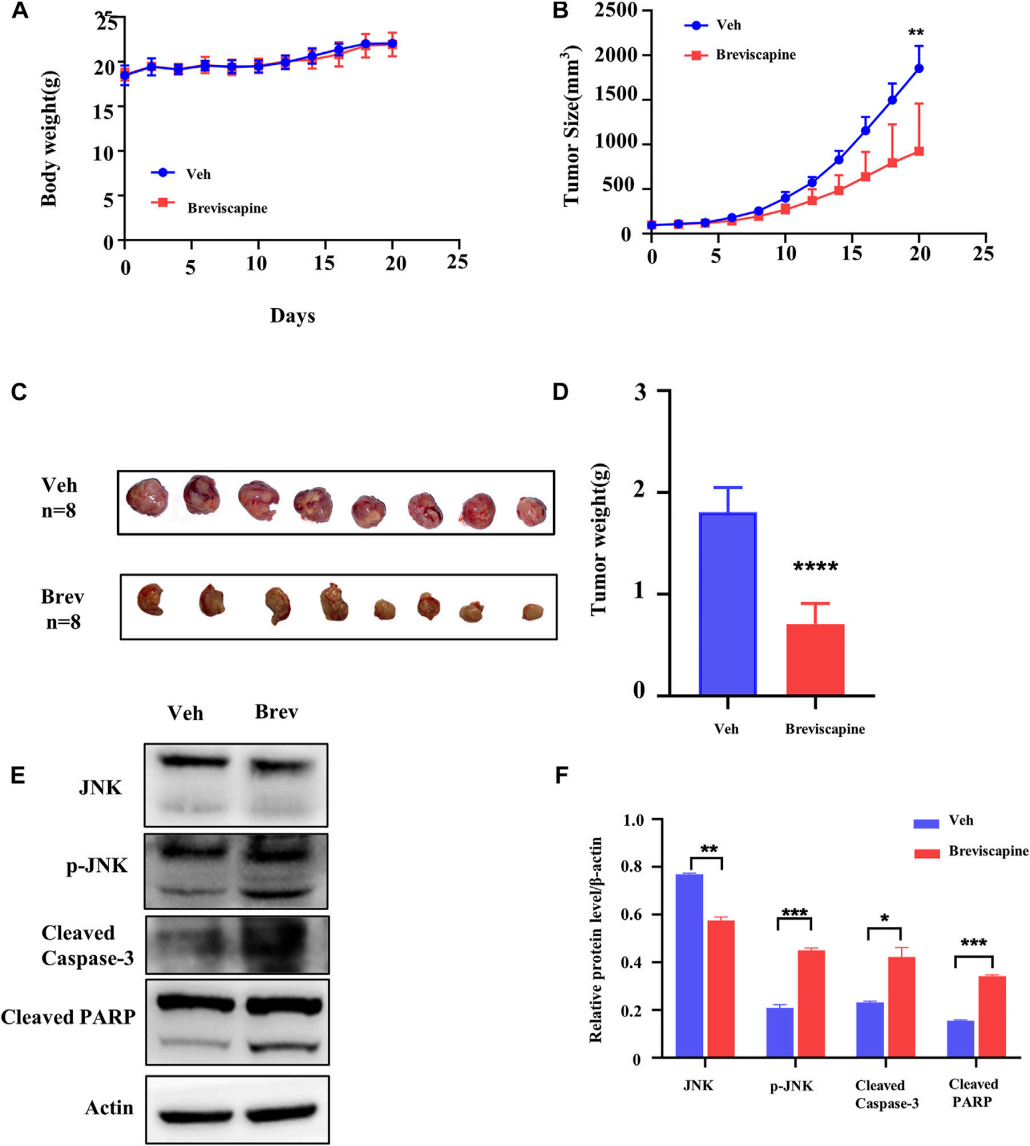
Scutellarin suppresses xenograft growth *in vivo* model. **(A)** The average body weight in breviscapine (60 mg/kg/d, po) and vehicle (normal saline, po) groups. **(B)** Xenograft size from nude mice in breviscapine and vehicle groups. **(C)** Xenograft excised from nude mice in each group. **(D)** The average weight of each xenograft. **(E)** The levels of JNK, p-JNK, cleaved Caspase-3 and cleaved PARP in the tumors with breviscapine treatment were evaluated by Western blotting. **(F)** The statistical analysis result of **(E)**. Veh n = 8, Brev n = 8. The data were shown as the mean ± SD, **p < 0.01, ****p < 0.0001.

According to network pharmacology analysis, the JNK/Caspase-3 pathway is the key signaling axis associated with the effect of scutellarin against AML. Thus, we analyzed the protein levels in this pathway by Western blotting. As shown in Figures 6F,G, all proteins including cleaved Caspase-3, p-JNK, and cleaved PARP showed an dose-dependent increase after scutellarin treatment. Furthermore, we found that JNK inhibitor SP600125 partially decreased the apoptosis (Figures 6H,I), reversed the protein levels of cleaved Caspase-3 and cleaved PARP (Figures 4J,K). Therefore, these findings indicated that scutellarin induced apoptosis in AML cells through the JNK/Caspase-3 pathway.

### Scutellarin suppresses xenograft growth *in vivo* model

Next, we explored whether scutellarin was still effective in the animal models of AML. HL-60 cells were inoculated subcutaneously into both hind limbs of nude mice, and breviscapine, containing scutellarin ≥90%, was administered at 60 mg/kg/d orally, which was corresponding to the maximum dose in humans per the guide for dose conversion between humans and mice (Nair & Jacob, 2016). Breviscapine did not affect the body weight of the mice (Figure 6A). But it inhibited the xenograft growth significantly in nude mice (Figures 6B–D). Subsequently, proteins from tumor tissues were extracted and subjected to Western blot analysis. Consistent with the *in vitro* findings, p-JNK, cleaved Caspase-3 and cleaved PARP were significantly increased (Figures 6E,F).

## Discussion

AML is a rapidly progressing hematopoietic malignancy whose global prevalence is rising. Although TCM has been proved to be a good choice for AML treatment, inferring the mechanism of action is challenging due to the complex chemical composition. The coincidence of the concept of network pharmacology and the theory of TCM makes the former suitable for the study of the mechanism of action of the latter (Luo et al., 2020).

This study employed a network pharmacology-based approach to infer the underlying molecular mechanism of scutellarin for treating AML, and also performed *in vitro* and *in vivo* experimental validation. First, we screened 289 potential targets for scutellarin from the PharmMapper database and retrieved 10998 AML-related genes from the OMIM, GeneCards, DrugBank, and PharmGkb databases. By merging targets of scutellarin with those of AML, a total of 253 overlapping genes were identified. Next, The results of PPI network analysis, GO and KEGG pathway analysis, revealed MAPK signaling pathway was critical for scutellarin-induced apoptosis. Thus, we chose the key proteins of MAPK and Caspase-3, a typical apoptosis-related protein for subsequent experimental validation.

The JNK signaling pathway regulates multiple cellular functions, such as apoptosis, survival, aging, cell proliferation, differentiation, and inflammation (Xu et al., 2015; Hammouda et al., 2020). It promotes apoptosis *via* the transcriptional regulation or phosphorylation of the key mitochondrial pro- and anti-apoptotic proteins (Dhanasekaran & Reddy, 2008). Modulation of the JNK pathway has been recognized as an effective avenue to treat cancers, including oral tumors, osteosarcoma, and colorectal cancer (Fu et al., 2020; Kon-Young Ji 2021; Liu et al., 2021), especially AML (Torres et al., 2010; Bomfim et al., 2019; Tian et al., 2019).

To verify the analysis of network pharmacology, we did *in vitro* and *in vivo* experiments. We found that scutellarin significantly induces apoptosis in AML cells. Additionally, Z-VAD-FMK, a specific inhibitor of apoptosis, significantly attenuated the pro-apoptotic effects of scutellarin. Accordingly, well-known apoptosis markers (Giuseppa Pistritto and claudia, 2016), such as cleaved PARP and cleaved Caspase-3, were induced by scutellarin dose-dependently. In addition, p-JNK was increased significantly with scutellarin treatment in HL-60 cells. Meanwhile, pretreatment with the JNK inhibitor SP600125 rescued scutellarin-induced apoptosis. Consistent with the *in vitro* data, scutellarin suppressed subcutaneous xenograft growth in nude mice, and the change of the protein levels in the JNK/Caspase-3 pathway was also recapitulated.

This study integrated network pharmacology-based prediction and experimental validation to uncover the importance of the JNK pathway in scutellarin-mediated AML treatment.

## Data Availability

The original contributions presented in the study are included in the article/Supplementary Material, further inquiries can be directed to the corresponding authors.

## Supplementary material

Supplementary Material S1 The target of Scutellarin predicted by PhamMapper.

Supplementary Material S2 The targets of AML predicted by OMIM, GeneCards, PharmGkb, rugBank.

Supplementary Material S3 Drug-Disease targets.

Supplementary Material S4 String-interactions.

Supplementary Material S5 Core genes.

Supplementary Material S6 GO.

Supplementary Material S7 KEGG.
